# SIRT1 and Autophagy: Implications in Endocrine Disorders

**DOI:** 10.3389/fendo.2022.930919

**Published:** 2022-07-14

**Authors:** Ji Yong Kim, David Mondaca-Ruff, Sandeep Singh, Yu Wang

**Affiliations:** Department of Pharmacology and Pharmacy, The University of Hong Kong, Hong Kong SAR, China

**Keywords:** SIRT1, autophagy, obesity, diabetes, cardiovascular disease, diabetic cardiomyopathy

## Abstract

Autophagy is a cellular process involved in the selective degradation and recycling of dysfunctional intracellular components. It plays a crucial role in maintaining cellular homeostasis and survival by removing damaged and harmful proteins, lipids, and organelles. SIRT1, an NAD^+^-dependent multifunctional enzyme, is a key regulator of the autophagy process. Through its deacetylase activity, SIRT1 participates in the regulation of different steps of autophagy, from initiation to degradation. The levels and function of SIRT1 are also regulated by the autophagy process. Dysregulation in SIRT1-mediated autophagy hinders the proper functioning of the endocrine system, contributing to the onset and progression of endocrine disorders. This review provides an overview of the crosstalk between SIRT1 and autophagy and their implications in obesity, type-2 diabetes mellitus, diabetic cardiomyopathy, and hepatic steatosis.

## Introduction

Autophagy is a highly conserved cellular process involved in cellular homeostasis by selectively degrading the nuclear and cytoplasmic organelles, lipids, and proteins (1). It is an adaptive process against metabolic stress by removing the damaged organelles, protein aggregates, harmful substances and recycling them for the synthesis of new cellular components (1). In mammals, 3 types of autophagy have been described, microautophagy, chaperone-mediated autophagy, and macroautophagy (2). During microautophagy, cargos are encapsulated and broken down by the direct invagination or protrusion of lysosome membrane (3). Chaperone-mediated autophagy does not involve membrane structures to engulf the proteins (3). It instead uses chaperones to identify a cargo protein that contains a pentapeptide motif (KFERQ) (4). The protein substrate is then unfolded and translocated across the lysosomal membrane for degradation (4). Macroautophagy involves the formation of a double-membrane vesicle known as autophagosome to sequester target cargo (1). The autophagosome sequestered-cargo is then degraded by the fusion between lysosome and autophagosome, which forms an autolysosome (1).

Apart from non-selective nutrient recycling, autophagy mediates the degradation of specific cargos, such as protein aggregates (aggrephagy/proteophagy), endoplasmic reticulum (reticulophagy/ER-phagy), mitochondria (mitophagy), peroxisome (pexophagy), nucleus (nucleophagy), pathogens (xenophagy), lipids (lipophagy), ribosome (ribophagy), or even lysosomes themselves (lysophagy) (5, 6). In mammals, selective autophagy is mediated by specific receptors, including squestosome1 (SQSTM1), neighbor of breast cancer type 1 (NBR1), Toll interacting protein (TOLLIP), B-cell lymphoma 2 (Bcl-2)/adenovirus E1B 19-kDa interacting protein-3 (BNIP3), optineurin (OPTN), FUN14 domain containing 1 (FUNDC1), prohibitin 2 (PHB2), reticulophagy regulator 1 (RETREG1), calcium-binding and colied-coil domain 2 (CALCOCO2), and nuclear FMR1 Interacting protein 1 (NUFIP1) (5, 6).

Autophagy is a dynamic process that is elegantly controlled by various signaling molecules, such as the mechanistic target of rapamycin (mTOR)-1, 5’ adenosine monophosphate-activated protein kinase (AMPK), protein kinase B (Akt), and sirtuin 1 (SIRT1) (7). This review focuses on macroautophagy (herein referred as autophagy) and will describe the current understanding on its crosstalk with the SIRT1 signaling pathways. The implications of the dysregulated SIRT1 function and autophagy in endocrine disorders, including obesity, type-2 diabetes mellitus, diabetic cardiomyopathy, and hepatic steatosis will be discussed.

## Process, Location and Regulation of Autophagy

**Figure 1:**
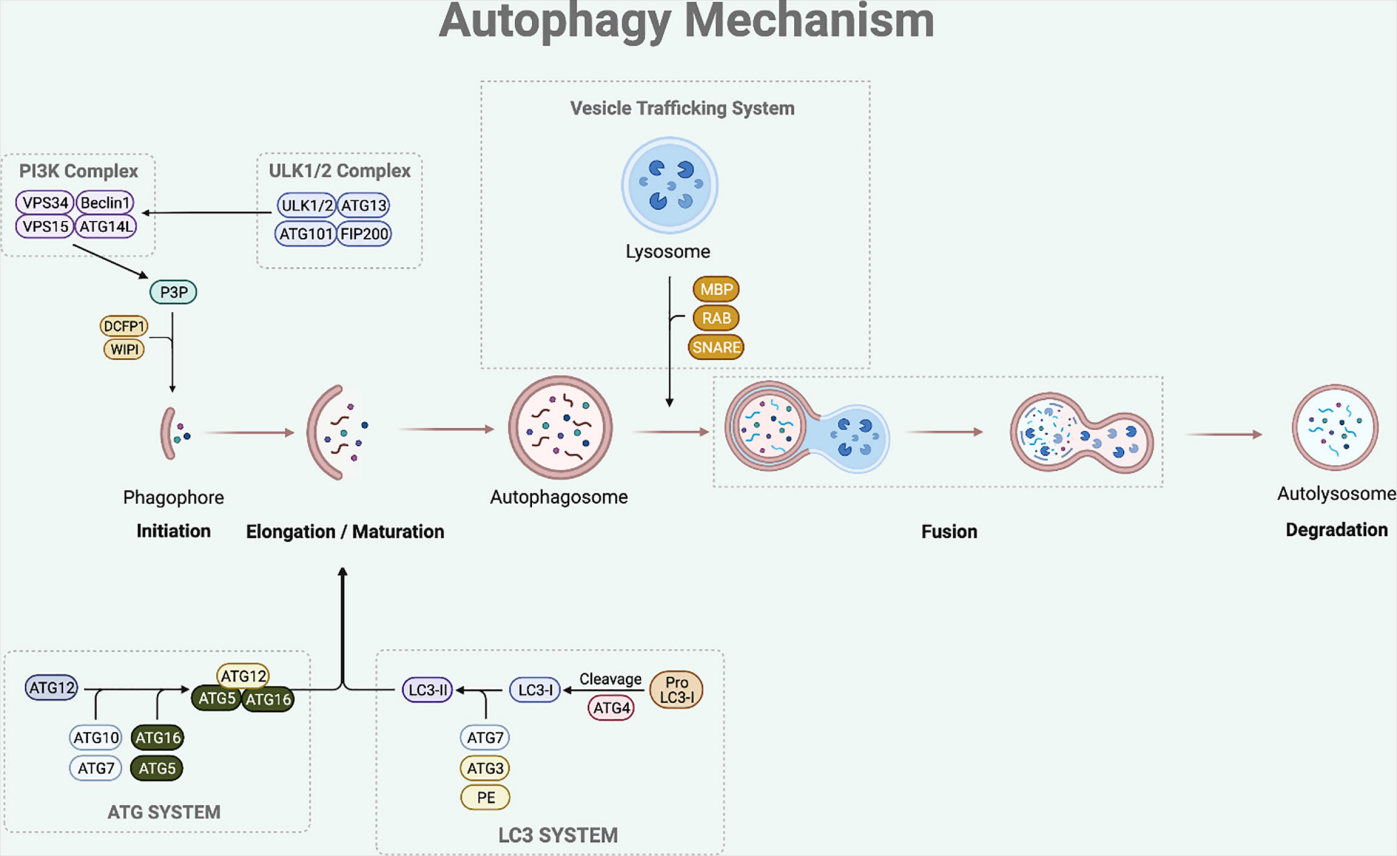
Autophagy Mechanism. The autophagy process involves multi-steps which are initiation, elongation, maturation, fusion and degradation. The initiation step of mammalian autophagy involves the ULK complex (consisting of ULK1/2, ATG13, FIP200, and ATG101). The ULK complex phosphorylates Beclin1 to activate PI3K complex (composed of Beclin1-VPS34-VPS15-ATG14L). The PI3K complex then induces P3P formation, which recruits DCFP1 and WIPI for autophagosome formation. During the elongation and maturation stage, ATG7 and ATG10 help to conjugate the ATG12 to ATG5, forming an ATG5-ATG12 complex. This complex then interacts with the ATG16 to form an ATG16-ATG5-ATG12 complex that is involved in elongation and maturation of the autophagosome. LC3-I is formed by the proteolysis of pro-LC3 by ATG4. The ATG3 and ATG7 conjugate LC3-I to PE to form LC3-II that inserts into the autophagosome membrane. After the elongation and maturation, autophagosome with target cargo is trafficked to the lysosome to fuse and form an autolysosome. This process is regulated by RAB protein family, SNARE protein family, and MBP. The autolysosome then releases acidic hydrolase to degrade the inner content and the degraded cargo is released back to the cytoplasm to be reused for cellular processes.

The sequential steps involved in autophagy process are initiation, elongation of phagophore, maturation of autophagosome, formation of autolysosome (fusion between autophagosome and lysosome), and degradation of the damaged or superfluous cellular components (8) (**Figure 1**).

The initiation step of mammalian autophagy involves the Unc51-like kinase (ULK) complex, which consists of ULK1/2, autophagy-related gene (ATG)13, FIP200, and ATG101 (9, 10). Under nutrient starved condition, the ULK complex dissociates from mechanistic target of rapamycin 1 (mTORC1) and translocates to autophagosome initiation site (11). At the initiation site, the ULK complex facilitates the activation of autophagy-specific class III phosphatidylinositol 3-kinase complex I (PI3KC3-CI), which consists of Beclin1, vacuolar protein sorting (VPS) 34, VPS15, and ATG14L (12). The PI3KC3-CI complex then leads to the formation of phosphatidylinositol-3-phosphate (P3P), which recruits double-FYV-containing protein 1 (DCFP1) and WD-repeat protein interacting with phosphoinositide (WIPI) for autophagosome formation at the characteristic endoplasmic reticulum (ER) structure known as the omegasome (13, 14).

Following the initiation process of autophagy, the elongation and maturation stages require the participation of two-ubiquitin like conjugation systems, which are the microtubule-associated protein 1 light chain 3 (LC3) and ATG system. ATG7 and ATG10 act in a similar manner to the E1 enzyme of the ubiquitin-proteasome system (15). They help to conjugate the ATG12 to ATG5, forming an ATG5-ATG12 complex (15). This complex then interacts with the ATG16 to form an ATG16-ATG5-ATG12 complex, which participates in the elongation and maturation of the autophagosome (16). During this step, LC3-I is produced by ATG4-mediated proteolysis of pro-LC3. The ATG3 (ubiquitin-like E2 enzyme) and ATG7 then conjugate LC3-I to phosphatidylethanolamine (PE) to form insoluble LC3-II that is inserted into the autophagosome membrane (13, 17). LC3-II is the hallmark of autophagic membrane formation and involved in the selective sequestration of the target cargo by recognizing and interacting with the cargo receptor protein (CRP), which then detects and binds to the LC3-interacting region (LIR) of the target cargo (18).

The final step of this dynamic process is fusion between autophagosome and lysosome leading to the degradation of the inner content. After the elongation and maturation step, autophagosome encapsulating the target cargo is trafficked to the lysosome to fuse and form an autolysosome. This process is regulated by the ras-associated binding (RAB) protein family, soluble N-ethylmaleimide-sensitive factor attachment protein receptor (SNARE) protein family, and membrane binding proteins (MBP) (15, 19). The autolysosome then releases acidic hydrolase to degrade the inner content and the degraded cargo is released back to the cytoplasm to be reused for cellular processes.

Autophagy mainly takes place in the cytoplasm, where autophagosome and autolysosome are formed (20). In response to an autophagy initiation signal, not only the autophagosome is nucleated at the cytoplasm but also LC3-I, which is primarily localized in the nucleus, is exported to the cytoplasm (20, 21). When autophagic initiation signal is given, the acetylated LC3-I localized in the nucleus is deacetylated by Sirtuin 1 (SIRT1) (17). The deacetylated LC3-I then binds to the nuclear diabetes and obesity regulated factor (DOR) protein, which helps LC3-I to export to the cytoplasm (17). At the cytoplasm, LC3-I is conjugated by the ATGs to form LC3-II, which helps sequestration of the target cargo for selective degradation. In addition to SIRT1, recent study by Shim et al. demonstrates the role of exportin-1 (XPO-1) as an alternative nuclear export of LC3-I (6). Under cyclic mechanical stress condition, the use of leptomycin B, XPO-1 dependent nuclear export inhibitor, led to the accumulation of LC3 in the nucleus (6). This LC3 accumulation in the nucleus was not observed when EX527, a SIRT1 inhibitor, was used (6).

Although the exact mechanism largely remains unclear, emerging evidences suggest that mammalian autophagy also plays a role in degradation of the components in the nucleus through a process known as nuclear autophagy. Nuclear autophagy is an evolutionarily conserved process in eukaryotes that helps to degrade nuclear components. It uses the nuclear envelop to target and encapsulate the autophagic substrate in nucleus, and exports them to the cytoplasm for lysosomal degradation (22). For example, under oncogenic condition, lamin B1 at the nucleus is recognized as an autophagic substrate and it directly interacts with the nuclear LC3 for autophagic degradation (23). Under senescent condition, nuclear SIRT1 also directly interacts with the nuclear LC3 for degradation (24–27). Under cyclic mechanical stress condition, nuclear LC3 interacts with NUFIP1 of ribosome and allow XPO-1 dependent nuclear-cytoplasmic transport for autophagic degradation (6).

**Figure 2:**
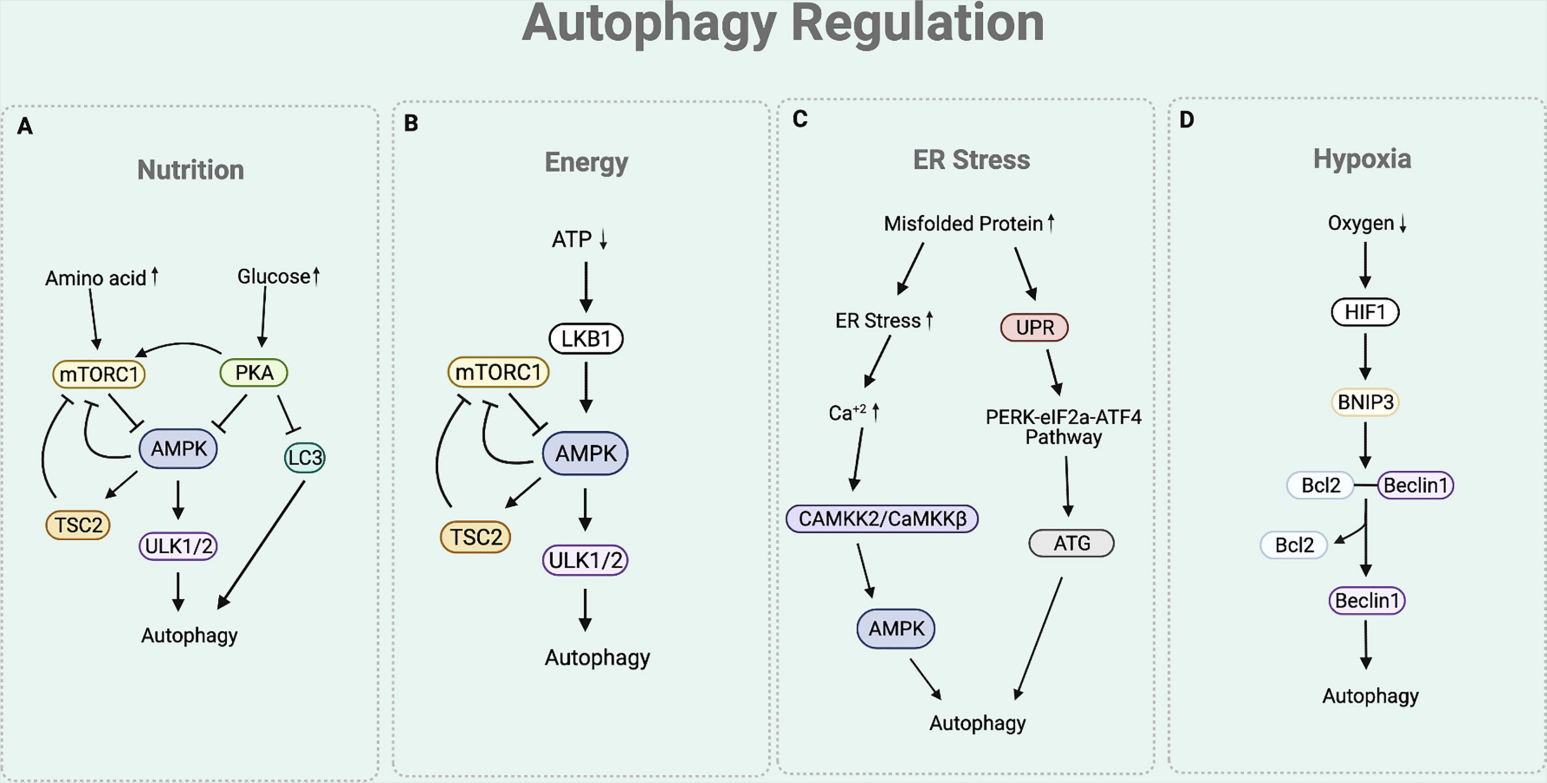
Autophagy Regulation. **(A)** The nutritional cue leads to the mTORC1 and cAMP-dependent-PKA-dependent pathways. In a nutrient rich condition (presence of amino acids), mTORC1 inhibits the interaction between ULK complex and AMPK by phosphorylating ULK1. Under nutrient depleted condition, mTORC1 is inactivated to allow the AMPK to interact with ULK1 for autophagy initiation. Under nutrition rich condition (presence of glucose), PKA inhibits autophagy process by phosphorylating LC3, activating mTORC1, and inactivating AMPK. **(B)** Under ATP depleted condition, LKB1 senses the decreased ATP/AMP ratio and activates AMPK, which inactivates mTORC1 by activating TSC2 and phosphorylating Raptor of mTORC1. This eventually leads to the upregulation of autophagy. **(C)** Under ER stress condition, the cytosolic Ca^+2^ level increases to activate CAMKK2/CaMKKβ that activates AMPK. AMPK helps to inhibit mTORC1 activity to induce autophagy by activating TSC2 and phosphorylating Raptor. The ER stress condition also triggers an UPR process that responds to an accumulation of unfolded proteins. UPR activates PERK-eIF2a-ATF4 pathway, which upregulates the production of ATGs necessary for autophagy process. **(D)** Under hypoxia condition, there is an increased expression of HIF1 that upregulates the expression of BNIP-3. BNIP3 promoter contains HRE that is activated by the HIF1. The BH3 functional domain of BNIP3 competitively interferes with the interaction between the Beclin1 and Bcl-2. This disruption liberates Beclin1 to be used for the autophagy initiation process.

As autophagy participates in maintaining cellular homeostasis, it is often regulated by various physiological stressors. Among these, the most studied autophagic signals are nutrition, energy, ER stress, and hypoxia (**Figure 2**) (28).

Nutritional cue regulates autophagy through the mTORC1 and cAMP-dependent protein kinase A (PKA)-dependent pathways (19) (**Figure 2A**). Under basal condition of eukaryotic cell, mTORC1 senses the presence of nutrients (19, 29). In a nutrient rich condition (mainly the presence of arginine, leucine, and glutamine), mTORC1 phosphorylates ULK1 at Ser 757, which disturbs the interaction between ULK complex and AMP-activated protein kinase (AMPK) (19). ULK complex-AMPK interaction is necessary for autophagy initiation as it helps AMPK to activate ULK complex by phosphorylating ULK1 at Ser 317 and 777 (11). Hence, under nutrient rich condition, activated mTORC1 prevents the interaction between ULK complex-AMPK, eventually inhibiting autophagy initiation (19). Similarly, PKA also participates in the downregulation of autophagy by sensing nutrition presence. Under glucose rich condition, PKA inhibits autophagy process by phosphorylating LC3 at Ser 12, directly activating mTORC1 (through phosphorylation at Ser 2448), and inactivating AMPK (29–31).

Presence of cellular energy, ATP, regulates autophagy through the Liver Kinase B1 (LKB1)-AMPK pathway (**Figure 2B**). Under ATP depleted condition, LKB1 senses the decreased ATP/AMP ratio and activates AMPK (32). In addition to the aforementioned direct activity of AMPK to induce autophagy by activating the ULK complex, AMPK also indirectly induce autophagy by inactivating mTORC1. Precisely, AMPK not only activates Tuberous Sclerosis Complex 2 (TSC2), a mTORC1 negative regulator, but also phosphorylates KIAA1303 (Raptor), a subunit of mTORC1, to prevent mTORC1 activity (32). As ATP depletion activates AMPK to directly and indirectly inactivate mTORC1, this eventually leads to the upregulation of autophagy.

Stress signal at the ER participates in the autophagy regulation (**Figure 2C**). The accumulation of misfolded proteins at the ER induces stress. Under ER stress condition, cytosolic Ca^2+^ level increases (29). This leads to the activation of calcium/calmodulin-dependent protein kinase kinase 2/β (CaMKK2/CaMKKβ) to activate AMPK (29). As the activity of AMPK is increased with ER stress, mTORC1 activity is inhibited while ULK complex is activated, consequently inducing autophagy. The ER stress condition also triggers the unfolded protein response (UPR) process, which facilitates the autophagy elongation machinery (33). Precisely, the ER membrane contains a UPR sensor that responds to an accumulation of unfolded proteins. UPR activates the double stranded RNA activated-protein kinase-like ER kinase (PERK)-eukaryotic translation initiation factor 2 (eIF2a)-activating transcription factor 4 (ATF4) pathway, which upregulates the production of ATGs necessary for autophagy process (33).

Hypoxia is another condition that is involved in the regulation of autophagy (**Figure 2D**). Under hypoxia environment, there is an upregulation of hypoxia inducible factor 1 (HIF1) (34). Increased expression of HIF1 upregulates the expression of BNIP3 as BNIP3 promoter contains a functional hypoxia response element (HRE) that is activated by HIF1 (34). The BH3 functional domain of BNIP3 competitively interferes with the interaction between the Beclin1 and Bcl-2 (35). This disruption liberates Beclin1 from Bcl-2 from the inhibitory complex, eventually leading it to be used for the autophagy initiation process (35).

## SIRT1 and Autophagy

SIRT is a family of nicotinamide adenine dinucleotide (NAD^+^)-dependent enzymes that play roles in diverse cellular processes including cell survival, metabolism, proliferation, senescence, apoptosis, and DNA repair (36, 37). All of the SIRT family members share NAD^+^-dependent catalytic domain, but they have varying localization and function due to the difference in the lengths and sequences of C and N terminal (36). In the mammals, there are 7 different isoforms of SIRTs (SIRT 1-7) that are sub-divided into 4 classes (class I-IV) according to the phylogenetic tree analysis (38). Apart from the well-known deacetylation activity, recent studies report that the mammalian SIRTs also exhibit other functions including ADP-ribosylation, demalonlysation, desuccinylation, and RNA polymerase I transcription machinery (37).

Class I consists of SIRT1-3 that participate in deacetylation. SIRT1 translocate between nucleus and cytoplasm, SIRT2 is expressed in cytoplasm, while SIRT3 is found in the nucleus and mitochondria (37). Class II includes SIRT4 that mainly functions as an ADP-ribosyl transferase in the mitochondria (37). Unlike Class I and II, Class III involves SIRT 5 and 6 that has various functions. SIRT5 is expressed in the mitochondria, acting as a deacetylase, demalonlyase, and desuccinylase (37). SIRT6 is located in the nucleus to regulate the nuclear ADP-ribosyl transferation and deacetylation (37). Lastly, Class IV includes SIRT7 that functions as deacetylase and RNA polymerase I transcription machinery in the nucleus (37).

Among the mammalian SIRTs, SIRT1 is the most studied and largest human SIRT, sharing closet homology with yeast silent information regulator (Sir2) (37). Human *SIRT1* gene consists of 11 exons with 33,715 base pairs and it locates at the 10q21.3 chromosome (39). The human *SIRT1* gene encodes 747-amino acid polypeptide, having conserved core domain with N-terminal and C-terminal extensions (37). The essential for SIRT1 activity (ESA) domain locates at the C terminal of SIRT1 (641-664 amino acids), and it interacts with the catalytic core domain for deacetylase activity (40–42). SIRT1 contains two nuclear localization signals (NLS), which extends from 32 to 39 and 223 to 230 amino acids, and two nuclear exportation signals (NES) that expands from 138 to 145 and 425 to 431 amino acids (42). These structures help dynamic translocation of SIRT1 between the nucleus and cytoplasm, allowing unique location-specific functions in different cells.

SIRT1 plays essential roles in regulating the autophagy process through its deacetylase activity (43, 44). SIRT1 mediates autophagy by interacting not only with the ATGs but also the upstream regulators that are involved in various steps of autophagy, which includes initiation, elongation, maturation, fusion, and degradation (45).

### SIRT1-Mediated Autophagy Initiation

**Figure 3:**
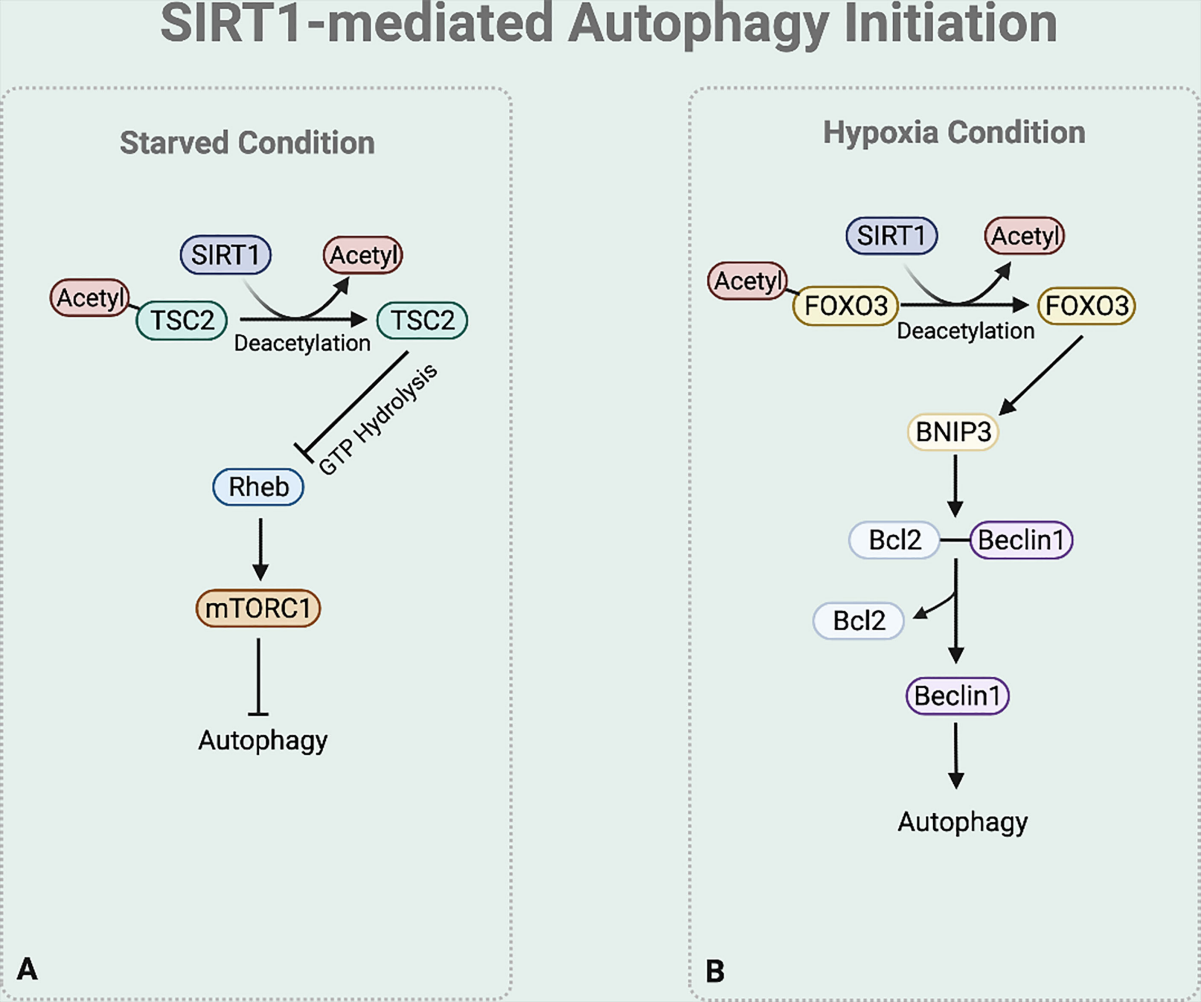
SIRT1-mediated autophagy initiation regulation. **(A)** Under starved condition, Deacetylase activity of SIRT1 also increases the stability of TSC2 to promote GTP hydrolysis of Rheb. Rheb suppression downregulates the mTOR signaling process. **(B)** Under hypoxia condition, SIRT1 deacetylases FOXO3 to bind to BNIP-3 promotor and promote BNIP-3 expression.

In autophagy initiation, SIRT1 regulates TSC2 stability (46) (**Figure 3A**). TSC2 suppresses the mTORC1 signaling pathway by inactivating Ras homolog enriched in brain (Rheb), which activates mTORC1. Rheb requires the binding of GTP for its activity to induce mTORC1 activation, while the GTPase-activating protein (GAP) domain of TSC2 stimulates GTP hydrolysis (47). Increased TSC2 stability increases GTP hydrolysis to inactivate Rheb, consequently downregulating the mTOR signaling process and initiating autophagy process. Under nutrition deprived condition, TSC2 complex stability is maintained by preventing ubiquitin-mediated degradation due to the deacetylase activity of SIRT1 (25, 28).

Moreover, emerging studies report that under hypoxia condition SIRT1 regulates the BNIP-3 mediated autophagy initiation (44) (**Figure 3B**). SIRT1 deacetylases the nuclear Forkhead box protein O3 (FOXO3) (48). This deacetylation leads FOXO3 to bind to BNIP-3 promotor, inducing BNIP-3 expression (48). The BNIP-3 expression then triggers autophagy by helping the dissociation of Beclin1 from the Bcl-2-Beclin1 complex (49). The released Beclin1 then activates PI3K complex which is necessary for autophagy initiation.

### SIRT1-Mediated Autophagy Elongation and Maturation

**Figure 4:**
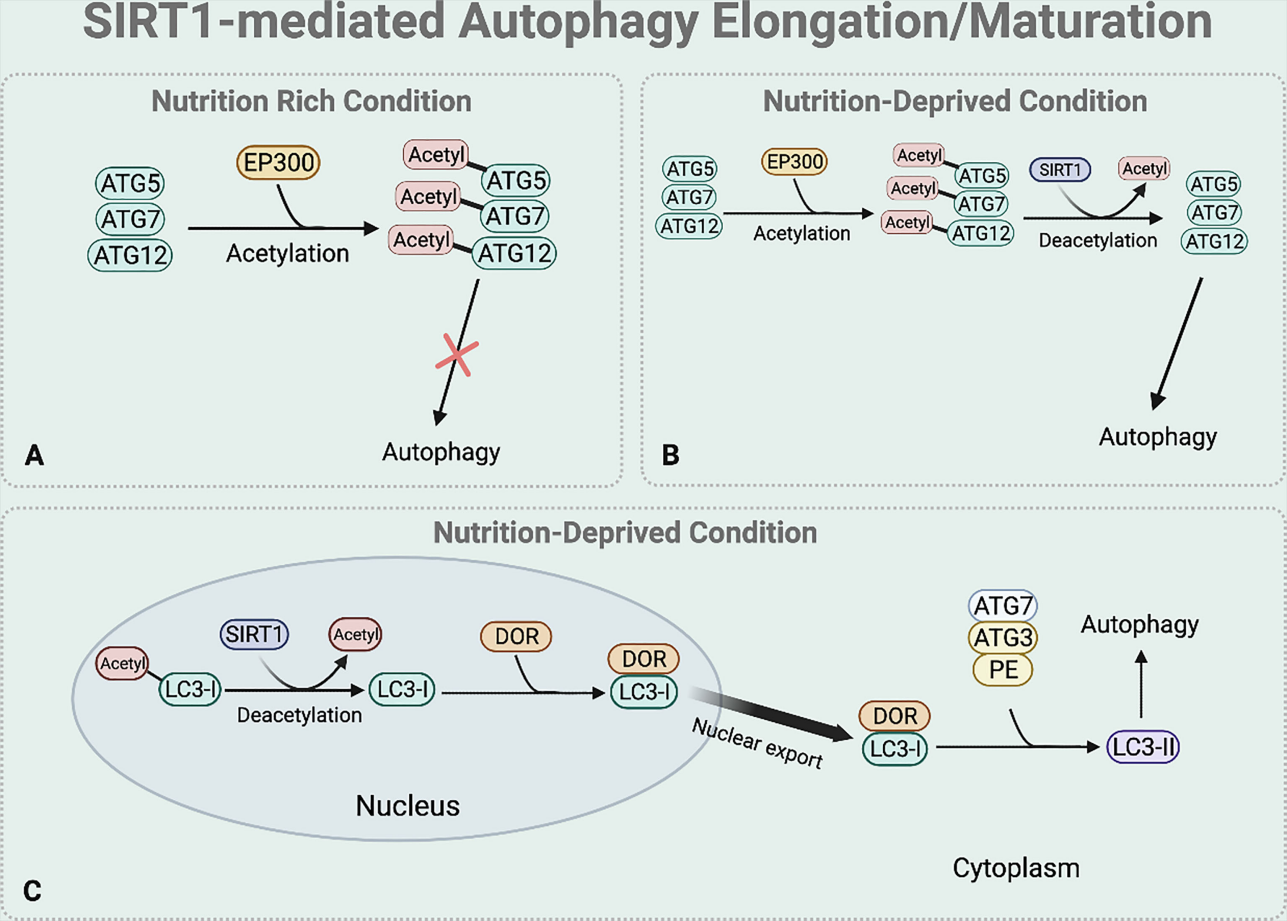
SIRT1-mediated autophagy elongation and maturation regulation. The BNIP-3 expression then induces autophagy by using its functional BH3 domain to dissociate Beclin1 from the Bcl-2-Beclin1 complex. **(A)** Under nutrient rich condition, EP300 acetyltransferase inhibits the elongation of the autophagosome by acetylating the ATG5, 7, and 12. **(B)** Under nutrient deprived state, SIRT1 directly deacetylates these ATGs to form ATG 16-5-12 complex that aids in the elongation of the autophagic vesicle. **(C)** Under starved condition, SIRT1 directly deacetylates the nuclear LC3-I. The deacetylated LC3-I then interacts with DOR protein and this interaction between the deacetylated LC3-I and DOR protein allows the nuclear export of LC3-I. The deacetylation of LC3-I also helps LC3-I to interact with ATG7. Through this interaction, the translocated LC3-I conjugates to PE, forming LC3-II which is inserted to the autophagosome membrane to help selective targeting of the cargo.

Elongation and maturation stages can be also regulated by the deacetylase function of SIRT1. Under nutrient rich condition, acetylation of ATG5, ATG7, and ATG12 by E1A binding protein p300 acetyltransferase (EP300) inhibits the formation of ATG16-ATG5-ATG12 complex, preventing autophagosome elongation (50) (**Figure 4A**). On the other hand, under nutrient starvation, SIRT1 facilitates the formation of ATG16-ATG5-ATG12 complex by directly deacetylating ATG5, ATG7, and ATG12, aiding elongation of the autophagic vesicle (51) (**Figure 4B**).

SIRT1 also regulates the translocation of nuclear LC3-I to the cytoplasm that is necessary for the formation of LC3-II which is inserted to the elongating autophagosome (44, 52) (**Figure 4C**). Under nutrient starvation, SIRT1 directly deacetylates nuclear LC3-I at Lys 49 and Lys 51 (44, 52). The interaction between the deacetylated LC3-I and DOR protein allows the nuclear export of LC3-I (44, 52). The deacetylated cytoplasmic LC3-I then interacts with ATG7, helping LC3-I to conjugate to PE and form LC3-II that is inserted to the autophagosome membrane for selective targeting of the cargo (44, 52).

### SIRT1-Mediated Autophagy Fusion and Degradation

**Figure 5:**
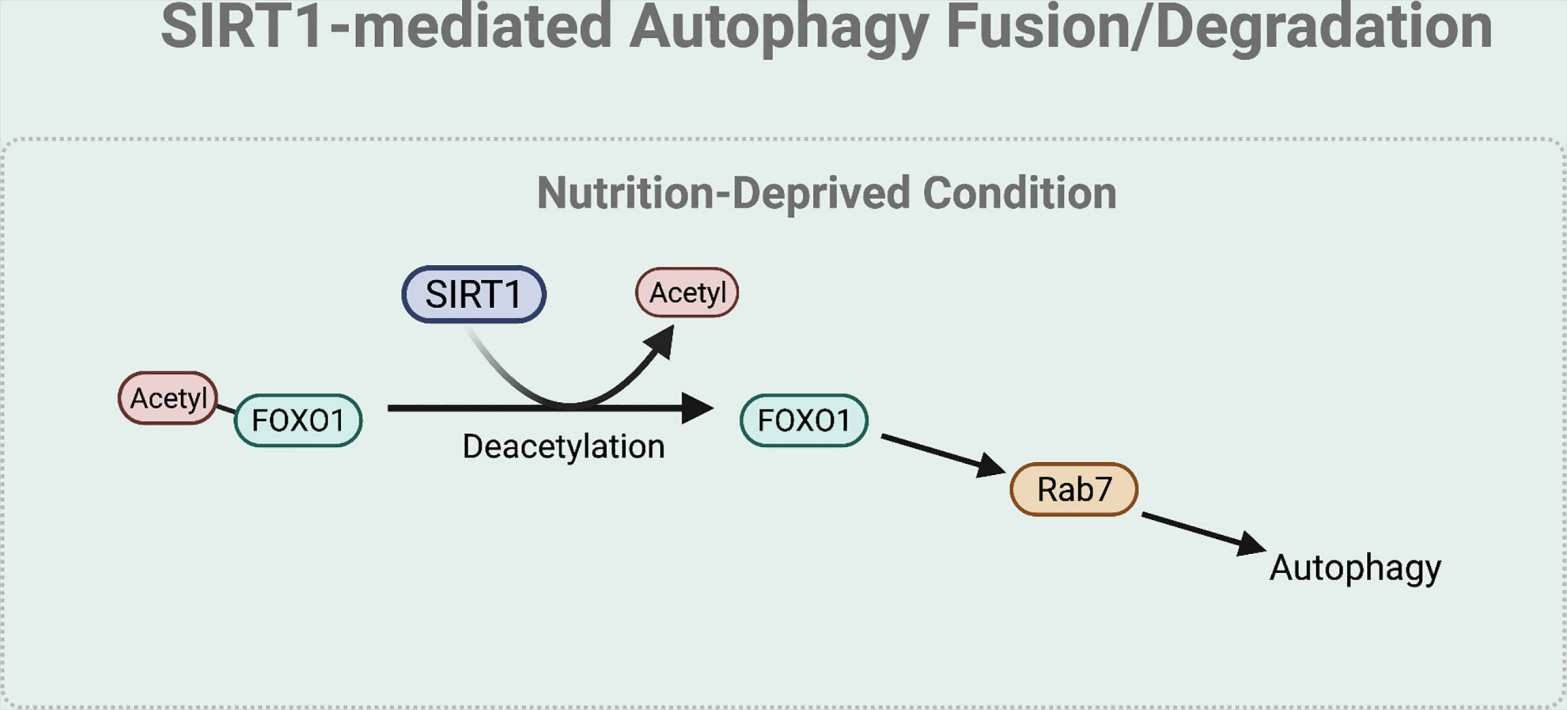
SIRT1-mediated autophagy fusion regulation. Under nutrient-starved condition, SIRT1 mediates the autophagy and lysosome fusion by deacetylating FOXO1. The deacetylation of FOXO1 is necessary for the expression of Rab7 that helps to regulate this microtubule-dependent bidirectional transport of the autophagosome to the lysosome.

The fusion and degradation process of autophagy can also be mediated by SIRT1 (**Figure 5**). Specifically, under nutrient starved condition, deacetylation of FOXO1 by SIRT1 regulates autophagosome-lysosome fusion by increasing expression of the Rab7. Rab7 participates in autolysosome formation by facilitating the trafficking of the mature autophagosome to the lysosome (45, 53). As most lysosomes are localized in the perinuclear region, autophagosome has to move towards the perinuclear region to fuse with the lysosome. Rab7 helps to regulate this microtubule-dependent bidirectional transport by interacting with LC3 (54).

### Dual Interaction Between SIRT1 and Autophagy

Emerging evidences demonstrate the dual interaction between SIRT1 and autophagy: not only SIRT1 regulates autophagic activity but also autophagy controls SIRT1 level by lysosomal degradation (26, 55). Depending on the cell condition, autophagy may upregulate or downregulate SIRT1 level. A recent study by Xu et al. suggests that under senescence condition, SIRT1 is recognized as a nuclear autophagy substrate (27). Through the direct interaction between LC3 and SIRT1, SIRT1 is transported to the cytoplasm for lysosomal degradation (26, 27). However, under non-senescent condition, autophagic activity is necessary in maintaining SIRT1 level. According to the study reported by Chen et al., endothelial cells treated with chloroquine, which is an autophagy blocker, showed significant decrease of SIRT1 level (56). Similar results were observed when the same cells were treated with 3-methyladenine (3-MA), an inhibitor of PI3K (56).

## SIRT1-Mediated Autophagy and Endocrine Disorders

Autophagy plays essential roles in tissue development and homeostasis by participating in the turnover of the intracellular components including lipids, proteins, and organelles (57). On the other hand, irregular autophagic process hinders the normal functioning of the metabolic process, leading to the induction of endocrine disorders (58). Accordingly, emerging evidences suggest that dysregulation in SIRT1-mediated autophagy is involved in the onset and development of obesity, type-2 diabetes mellitus, and diabetic cardiomyopathy.

### SIRT1-Mediated Autophagy and Obesity

Obesity is a complex endocrine disorder characterized by an excessive fat accumulation in adipose tissue of an individual (59). Autophagy is closely associated to obesity as abnormal changes in autophagic activity disturbs the normal lipid homeostasis, contributing to the increased fat content in the adipose tissue (60). The role of autophagy in obesity is shown in a recent study by Zhang et al. demonstrating that inhibition of autophagy through the downregulation of FOXO3 leads to lipid accumulation in adipocytes of mice (61). The association between autophagy inhibition and adipocyte lipid accumulation was also described in a study by Wu et al. The use of icaritin, an osteoinductive agent, attenuated lipid accumulation with an increase in autophagy flux (62). As adipocyte lipid accumulation is closely related to obesity, this suggests that autophagy may participate in regulating obesity condition by inhibiting lipid accumulation. Similarly, this association between autophagy and obesity is recently reported by Nunzio et al. in human studies, where they demonstrated that obese patients have significantly lower levels of LC3 in compared to the normal weight (63).

SIRT1 dysregulation in adipose tissue disturbs autophagic activity to degrade lipid molecules, leading to lipid droplets accumulation in the adipocytes and contributing to the development of obesity. The link between SIRT1-mediated autophagy and obesity was recently demonstrated by a study by Li et al. in an adipose-specific SIRT1 knockout mice. The SIRT1 knockout mice exhibited an increase in the fat mass and body weight with an increased exosome, which is due to the defect in autophagy activity (reduced LC3-II and ATG7 level) (64). Restoring SIRT1 activity with the SIRT1 activator, SRT1720, improved autophagy impairment and metabolic abnormality.

Other than changes in the lipid homeostasis, it has been previously reported that reduced energy expenditure and locomotor activity are another major influence leading to obesity (65, 66). In relation to this, Meng and Cai described that autophagy defects in the hypothalamus plays a role in the development of obesity through reduced energy expenditure and locomotor activities. According to their study, high-fed diet mice with medisobasal hypothalamic region-directed ATG7 knockdown not only developed much more severe obesity but also showed reduced energy expenditure and locomotor activities (67). Recent study by Xiao et al. further suggests the link between autophagy and hypothalamic obesity. High-fed diet mice with ATG5 knock-out in proopiomelanocortin (POMC) neurons, which are located at the arcuate nucleus of the hypothalamus, showed both reduced energy expenditure and increased fat mass (68). In addition, study by Skobo et al. suggests that impairment of the autophagic process in skeletal muscles also reduces locomotor activity thereby reducing energy expenditure and inducing obesity. Knockdown of Ambra1, which is an essential component of Beclin1, impaired the locomotor activity and skeletal muscle development in Zebrafish (69). Hence, these overall suggest that autophagy impairment may not only be associated to obesity development due to changes in lipid homeostasis but also reduced locomotor activities and energy expenditure.

SIRT1 dysregulation in hypothalamus and skeletal muscle may also be associated to the development of obesity. In relation to the aforementioned link between autophagy impairment in the hypothalamus and obesity, Cakir et al. demonstrated that changes in the SIRT1-FOXO1 axis in hypothalamus is also associated to obesity. Rats with either pharmacological inhibition or siRNA-mediated knock down of hypothalamic SIRT1 showed significant increase in body weight and food intake than control (70). The decreased level of acetylated FOXO1 suggested that SIRT1 regulates body weight in FOXO1 dependent manner (70). As autophagy is regulated through the SIRT1-FOXO1 axis and autophagy impairment in hypothalamus also leads to obesity, this suggests that SIRT1-FOXO1-autophagy axis in hypothalamus may be also associated to the development of obesity. Similarly, study by Ryall et al. suggested that decrease in SIRT1 level in skeletal muscle leads to disruption of muscle regeneration, development, thereby impairing the motor activity due to a mechanism that involve a decrease in NAD^+^ level (71). Price et al. also reported that increased SIRT1 improves in mitochondrial function in skeletal muscles through an AMPK pathway thereby increasing energy expenditure (69, 72). As the stimulation of AMPK increases autophagy activity, this suggests a close relationship between SIRT1 and autophagy in skeletal muscle function and development, consequently improving skeletal function and energy expenditure to protect against obesity.

### SIRT1-Mediated Autophagy and Type-2 Diabetes Mellitus

Type-2 diabetes mellitus (T2DM) is a condition characterized by high blood glucose level, insulin resistance, and lack of insulin secretion. These processes are normally related to impaired insulin sensitivity and pancreatic *β*-cell function to produce or secrete insulin to the blood. Under non-pathological condition, autophagy participates in improving insulin sensitivity and *β*-cells survival.

Insulin sensitivity is associated with activities of hypothalamus, skeletal muscle, liver, and adipose tissue, which are insulin-responsive (73). These insulin-responsive tissues mediate glucose uptake process depending on the presence of insulin. Yamamoto et al. recently demonstrated that autophagy participates in the insulin signaling process of liver, skeletal muscle, and adipose tissue using mouse model with increased constitutive autophagy by Beclin1 mutation. Under high food diet, this mouse exhibited enhanced insulin sensitivity at skeletal muscle, liver, adipose tissue than mouse with basal levels of autophagy by reducing ER stress (74, 75). These effects were reversed after the use of the autophagy inhibitor SBI-0206965 (74, 75). In addition, Meng and Cai previously reported that hypothalamic autophagy is also necessary in systemic insulin sensitivity by using chronic high fat diet mice. The mice with medisobasal hypothalamic region-directed ATG7 knockdown developed systemic insulin resistance with higher level of glucose intolerance and hyperinsulinemia, suggesting that autophagy is necessary in maintaining insulin sensitivity (67).

Moreover, the role of autophagy in *β*-cell survival is demonstrated by a study by Liu et al. According to the study, intermittent fasting in mice fed with high fat diet improved glucose tolerance *β*-cell function, and *β*-cell mass by restoring autophagic flux (76). However, this improvement in glucose tolerance was not evident when same condition was applied to the autophagy defective mice (76). Similar results were also observed previously in the study by Bartolomé et al. The mice with decreased autophagic activity due to *β*-cell specific deletion of TSC2 showed increased *β*-cell apoptosis and *β*-cell function failure (77). Hence, the aforementioned findings overall suggest that autophagy plays a crucial role in preventing T2DM by improving insulin sensitivity and pancreatic *β*-cell function.

As autophagy is necessary in preventing T2DM by improving insulin sensitivity and pancreatic *β*-cell function, dysregulation in SIRT1-autophagy axis may hinder the normal autophagy activity, eventually leading to the onset and exacerbation of T2DM condition. The link between SIRT1-mediated autophagy and T2DM is described in a recent study by Ren et al., demonstrating that metformin treatment attenuated fat diet induced-T2DM condition in a rat model with elevated levels of SIRT1, Beclin1, ATG12, LC3, and FOXO1. These findings suggest that SIRT1-FOXO1-autophagy mechanism participates in the process of T2DM condition (78). The link between SIRT1-autophagy-T2DM axis is further supported by a study performed by Ma et al. According to the study, SIRT1 knockout rats not only showed less amelioration of the T2DM condition with resveratrol treatment but also showed a decrease in the autophagy markers (ATG5, ATG7, and LC3) (79). A possible mechanism underlying the SIRT1-autophagy-T2DM was recently demonstrated by Josephrajan et al. Fatty acid binding protein 4 (FABP4) is a protein that is expressed by the adipocytes and it is necessary in insulin sensitivity. SIRT1 knockout mice exhibited decreased FABP4 secretion, similar to the results when ULK1/2 and VPS34 were inhibited (80). This suggests that FABP4 is secreted from adipose tissue in SIRT1-dependent manner *via* mechanism that requires autophagic components (80). Dysregulation of the SIRT1-autophagy axis may hinder FABP4 expression leading to insulin sensitivity impairment and progression of T2DM. Hence, these findings overall illustrate the link between SIRT1-mediated autophagy and T2DM.

### SIRT1-Mediated Autophagy and Diabetic Cardiomyopathy

Diabetic cardiomyopathy is commonly referred as an irregular cardiac function and structure in diabetic individuals with an absence of other cardiac risk factors such as coronary artery disease and endothelial senescence (81). Autophagy is an essential cellular mechanism that is involved in diabetic cardiomyopathy development as it not only participates in regulating diabetes-inducing factors but also cardiac functions. A recent study done by Zang et al. demonstrates the link between autophagy and diabetic cardiomyopathy. In a diabetic mouse model, cardiac autophagy inhibition through the knockout of ATG5 led to early onset and accelerated progress of the cardiomyopathy condition (82). Similarly, it has been previously described by Sciarretta et al. that autophagy activation helps to alleviate myocardial ischemia in diabetic mice. Inhibition of autophagy by ATG7 depletion and mTORC1 activation increased infarct size and cardiomyocyte death (83). On the other hand, restoration of autophagy activity through mTORC1 inhibition and ATG7 re-expression reduced cardiomyocyte death and infarct size (83). These studies demonstrate that autophagy is associated to diabetic cardiomyopathy by alleviating the cardiac structural and function dysfunction under diabetic condition.

Emerging evidence suggests the role of SIRT1-mediated autophagy in diabetic cardiomyopathy. Ma et al. described that cardiac-specific SIRT1 knock out demonstrates diabetic cardiomyopathy symptoms including cardiac hypertrophy, insulin resistance, and irregular glucose metabolism (84). Furthermore, results from a recent study by Ren et al. suggested that modulating the SIRT1-FOXO1 mediated pathway helps to improve diabetic cardiomyopathy condition (85). In high-fat diet-induced diabetic rats, treatment of curcumin, which is a SIRT1 activator, decreased FOXO1 acetylation and improved myocardial function by decreasing apoptosis of cardiomyocytes (85). Similar results were also observed in a study by Makino *et al*, in which caloric restriction of diabetic rat showed improvement in left ventricular diastolic function with elevated level of SIRT1, FOXO1, and autophagy markers (LC3-II and Beclin1) (86). Overall, these findings suggest that SIRT1-mediated autophagy participates in alleviating diabetic cardiomyopathy condition.

### SIRT1-Mediated Autophagy and Hepatic Steatosis

Hepatic steatosis, known as fatty liver disease, is characterized by excess accumulation of intrahepatic fat. Chronic hepatic lipid accumulation induces adverse effect on the liver such as liver inflammation and metabolic dysfunction (87). Emerging studies have reported that autophagic process may be associated to the development of hepatic steatosis. The link between autophagy and hepatic steatosis is described in the study by Chang et al. using an obese and diabetic Otsuka Long-Evans Tokushima Fatty (OLETF) rat models. When the rats were administered with ezetimibe, they not only showed significant decrease in triglycerides, free fatty acids, and total cholesterol levels but also showed a significant increase in autophagy markers including ATG5, ATG6, ATG7 and LC3 (88). This ezetimibe-mediated improvement in hepatic steatosis with increase autophagy activity suggests that increase in autophagic activity may alleviate hepatic steatosis. However, recent study by Lima et al. reports that unlike mice autophagic activity may be associated in progression of hepatic steatosis in humans. Knockdown of ATG3 in human hepatocyte ameliorated hepatic steatosis condition while overexpression of ATG3 increased lipid load in hepatocytes (89). This renders possibility that autophagic model may function differently depending on the organism. Also, although all ATGs are essential in autophagic machinery, in the context of liver steatosis, each ATG may have different role in the autophagy-hepatic steatosis interaction.

The link between SIRT1 and hepatic steatosis has been previously reported by Li et al. Liver-specific SIRT1 knock out mice showed severe hepatic steatosis compared to the control (90). In relation to this, accumulating studies demonstrate that SIRT1 may influence the progression of hepatic steatosis in an autophagy mediated manner. The study by Song et al. describes that metformin alleviates hepatosteatosis by restoring SIRT1-mediated autophagy induction. Obese mice with metformin treatment showed improved hepatic steatosis with upregulation of SIRT1 expression and LC3-II level (91). Metformin also decreased lipid accumulation in the liver and prevented fatty acid-induced suppression of SIRT1-dependent activation of autophagy process (91). Recent study by Ren et al. supports the association between the SIRT1-autophagy axis and hepatic steatosis. Compared to control, both rapamycin (autophagy inhibitor) and SRT1720 (SIRT1 activator) showed significant improvement in hepatic steatosis condition of high-fat diet mice (92). In addition, melatonin treatment enhanced autophagy activity with improvement in hepatic steatosis condition (92). However, when SIRT1 was inhibit in melatonin-treated mice, expression level of melatonin-induced autophagy related genes and melatonin-induced protective effect on liver decreased (92). Similar to melatonin, the link between SIRT1-mediated autophagy and hepatic steatosis was observed in a study by Hong et al. using erythropoietin. Mice that received erythropoietin showed increased level of SIRT1 and LC3 with alleviating hepatic steatosis (93). However, when erythropoietin was given to SIRT1 knockout mice, not only erythropoietin-induced LC3 level but also hepatic steatosis condition decreased (93). Hence, these overall suggest that SIRT-mediated autophagy may be associated to the hepatic steatosis to a certain extent.

## Potential Therapeutic Intervention

As the role of SIRT1-mediated autophagy in metabolic disorders have been increasingly highlighted, clinical and animal studies have been conducted to validate the use potential SIRT1 modulator as a therapeutic intervention for endocrine disorders by targeting SIRT1-autophagy axis.

Accumulating studies have shown that resveratrol acts as a SIRT1 activator (72, 79, 84, 94–97). In addition, clinical studies report that resveratrol have beneficial effects on patients with endocrine disorders such obesity and hepatic steatosis. Precisely, the effect of resveratrol on obesity and hepatic steatosis has been described in a clinical study done by Timmers et al. Obese patients, who received 150 mg of resveratrol per day for 30 days, have shown increased plasma levels of SIRT1 (98). Moreover, resveratrol treatment decreased intrahepatic lipid content, plasma fatty acid, glycerol, and triglyceride level (98). Overall, 30 days of resveratrol treatment mimicked the effect of caloric restriction in obese humans. Recent clinical study by Hoseini et al. suggests that resveratrol also has a beneficial effect on patients with diabetes. Diabetic patients who received 500mg of resveratrol per day for 4 weeks showed significant decrease in insulin resistance, glucose, and cholesterol level while increasing insulin sensitivity (99). Resveratrol also increased T2DM patients’ SIRT1 level (99).

Although the mechanism underlying the effect of resveratrol on endocrine disorder in humans remain unclear, *in vivo* studies describe that resveratrol may target the SIRT1-autophagy axis to improved disease condition. Ding et al. demonstrated that resveratrol may target SIRT1-mediated autophagy pathway to prevent hepatic steatosis and obesity using eight-week old male Wistar rats. Rats treated with resveratrol not only showed partial prevention of hepatic steatosis symptoms with increased SIRT1 and autophagy markers level, but also decreased energy intake and body weight (100). The effect of resveratrol on SIRT1-autophagy axis to ameliorate diabetic cardiomyopathy was also shown in a study by Wang et al. using a diabetic mice model. Long term resveratrol not only improved cardiac function of but also reduced apoptosis in diabetic mouse heart (101). Resveratrol also increased SIRT1, FOXO1, Rab7, and autophagy flux with enhanced FOXO1 binding at Rab7 promoter region (101). On the other hand, SIRT1 and Rab7 siRNA prevented the resveratrol improvement of autophagy flux and cardiac function, suggesting that resveratrol participates in the improvement of diabetic cardiomyopathy through SIRT1-FOXO1-Rab7-autophagy pathway (101). Therefore, the clinical and *in vivo* findings overall suggest resveratrol as a potential therapy which targets SIRT1-mediated autophagic pathway to treat endocrine disorders.

In addition to resveratrol, recently several studies have reported the beneficial effect of NAD^+^ supplements in preventing endocrine disorders by increasing SIRT1 levels and activity. A clinical study by Yoshino et al. suggests that nicotinamide mononucleotide (NMN), which is a rate-limiting factor in mammalian NAD^+^ biosynthesis, helps to improve muscle insulin sensitivity of obese women (102). Moreover, Weijer et al. reports that nicotinic acid derivative acipimox, an NAD^+^ precursor has beneficial effect on skeletal muscle mitochondrial function (103). Improving mitochondrial function in skeletal muscle improves energy expenditure, which helps to improve obese condition. Moreover, Hsieh et al. reports that NAD^+^ supplement may enhance autophagy. Endothelial cells treated with nicotinamide, which increases the intracellular NAD^+^, showed increased level of LC3-II and autophagosome formation (104). This link between NAD^+^ supplement and autophagy suggest a possible mechanism by which increased NAD^+^ levels may activate SIRT1 to enhance autophagy, consequently inducing beneficial effects on the aforementioned endocrine disorders.

## Concluding Remarks

Given the essential role of autophagy in cell homeostasis, the functional activity and regulation of autophagy have been extensively studied in recent years. In this review, we described the autophagy machinery and the role of SIRT1 in regulating autophagy at different stages ranging from initiation to the degradation process. Through its deacetylase characteristics, SIRT1 can either directly act on the autophagy machinery or indirectly alter the upstream regulators to regulate autophagy activity. Moreover, the SIRT1-autophagy-endocrine disorder interplay indicates that SIRT1-mediated autophagy may play a key role in the onset and progression of endocrine disorders. Studies suggest that dysregulation of SIRT1-mediated autophagy may participate in the development of obesity, T2DM, diabetic cardiomyopathy, and hepatic steatosis.

However, most studies have only demonstrated the association between SIRT1-mediated autophagy and endocrine disorders. This leads to several unanswered questions about the underlying molecular mechanism on SIRT1-autophagy-endocrine disorder axis. Given that SIRT1 can regulate different steps of autophagy process, it still remains unclear through which mechanism SIRT1 regulate autophagy to induce endocrine disorder. Moreover, studies have revealed potential SIRT1-autophagy modulators such as icaritin and curcumin. However, the underlying molecular mechanism still remains unclear and the clinical studies are lacking to validate their effect on endocrine disorders in humans. Conducting these studies will provide a valuable insight on novel therapeutic targets for endocrine disorders. Lastly, studies have reported that SIRT1-mediated autophagy not only play a role in endocrine disorders but also other diseases such as neurodegeneration and nephropathy (79, 105). Investigating the implication of SIRT1-mediated autophagy in these diseases may be new frontiers for exploration which may also provide new pharmacological targets.

## Author Contributions

JYK, DM-R, and SS drafted the manuscript and designed the figures. YW supervised the students and revised and approved the final version of the review. All authors contributed to the article and approved the submitted version.

## Funding

This work was supported by the grants from Seeding Funds for Basic Research of the University of Hong Kong, the General Research Funds (17153016, 17124718, 17124420) and Collaborative Research Funds (C7037-17W) of Research Grant Council, the Areas of Excellence Scheme (AoE/M-707/18) of University Grants Committee, Hong Kong SAR.

## Conflict of Interest

The authors declare that the research was conducted in the absence of any commercial or financial relationships that could be construed as a potential conflict of interest.

## Publisher’s Note

All claims expressed in this article are solely those of the authors and do not necessarily represent those of their affiliated organizations, or those of the publisher, the editors and the reviewers. Any product that may be evaluated in this article, or claim that may be made by its manufacturer, is not guaranteed or endorsed by the publisher.
